# Identification, Characterization, and Heritability of Murine Metastable Epialleles: Implications for Non-genetic Inheritance

**DOI:** 10.1016/j.cell.2018.09.043

**Published:** 2018-11-15

**Authors:** Anastasiya Kazachenka, Tessa M. Bertozzi, Marcela K. Sjoberg-Herrera, Nic Walker, Joseph Gardner, Richard Gunning, Elena Pahita, Sarah Adams, David Adams, Anne C. Ferguson-Smith

**Affiliations:** 1 Department of Genetics, University of Cambridge, Cambridge CB2 3EH, UK; 2 Experimental Cancer Genetics, Wellcome Trust Sanger Institute, Hinxton, Cambridge CB10 1SA, UK

## Abstract

Generally repressed by epigenetic mechanisms, retrotransposons represent around 40% of the murine genome. At the *Agouti viable yellow* (*A*^*vy*^) locus, an endogenous retrovirus (ERV) of the intracisternal A particle (IAP) class retrotransposed upstream of the *agouti* coat-color locus, providing an alternative promoter that is variably DNA methylated in genetically identical individuals. This results in variable expressivity of coat color that is inherited transgenerationally. Here, a systematic genome-wide screen identifies multiple C57BL/6J murine IAPs with *A*^*vy*^ epigenetic properties. Each exhibits a stable methylation state within an individual but varies between individuals. Only in rare instances do they act as promoters controlling adjacent gene expression. Their methylation state is locus-specific within an individual, and their flanking regions are enriched for CTCF. Variably methylated IAPs are reprogrammed after fertilization and re-established as variable loci in the next generation, indicating reconstruction of metastable epigenetic states and challenging the generalizability of non-genetic inheritance at these regions.

## Highlights

• Repertoire of variably methylated repeat elements defined in inbred mice
• VM-IAPs are flanked by CTCF binding sites, and very few act as promoters
• Methylation variability is re-established from one generation to the next
• Memory of parental methylation state is an exception rather than the rule

## Graphical Abstract

**Figure:**
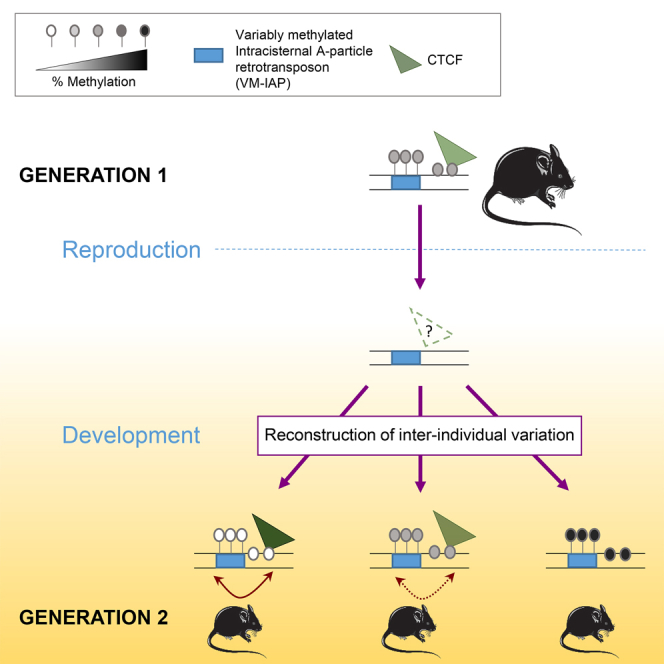

## Teaser

A genome-wide interrogation of variably methylated endogenous retroviruses shows common reprogramming of methylation states after fertilization, challenging the paradigm of transgenerational non-genetic inheritance at such loci.

## Introduction

Most interindividual phenotypic variation is explained by genetic variation. However, studies in plant and animal models indicate that non-genetic mechanisms can contribute to phenotypic variability, and such phenotypes can be inherited over multiple generations (Cubas et al., 1999, Morgan and Whitelaw, 2008, Becker and Weigel, 2012). Epigenetic changes in the absence of genetic effects have been reported to have long-lasting phenotypic outcomes over multiple generations in non-mammalian organisms. In mammals, such non-genetic effects are difficult to explain mechanistically, and it has been challenging to define the regulatory processes underlying the observed phenomena (Miska and Ferguson-Smith, 2016).

Two of the best-characterized paradigms of non-genetic inheritance in mammals occur at the murine *Agouti viable yellow* (*A*^*vy*^) and *Axin Fused* (*Axin*^*Fu*^) loci (Dickies, 1962, Vasicek et al., 1997). In these naturally occurring mutant mice, genetically identical individuals exhibit quantifiable phenotypic variability in coat color or tail morphology due to the insertion of an endogenous retrovirus (ERV) of the intracisternal A particle (IAP) class into the *Agouti* or the *Fused* loci, respectively. The range of phenotypes correlates reproducibly with interindividual differences in the level of DNA methylation at a long terminal repeat (LTR) promoter of the IAP, driving abnormal expression of the genes (Michaud et al., 1994, Rakyan et al., 2003). The consistency in methylation level observed within an individual is in contrast to the variation of methylation levels and phenotypic outcomes observed between individuals, defining *A*^*vy*^ and *Axin*^*Fu*^ as so-called “metastable epialleles” (Rakyan et al., 2002). Transgenerational inheritance of the methylation pattern at these metastable epialleles has been observed, whereby the distribution of phenotypes in the offspring was shown to be dependent on parental phenotype (Morgan et al., 1999, Rakyan et al., 2003). Furthermore, *A*^*vy*^ is susceptible to environmental influence impacting methylation and phenotype (Wolff et al., 1998, Dolinoy et al., 2006, Dolinoy et al., 2007, Kaminen-Ahola et al., 2010). Using genetic screens, proteins with epigenetic function associated with the maintenance of *A*^*vy*^ have been identified (Daxinger et al., 2013). In another study, a C57BL/6J endogenous IAP insertion at *Cdk5rap1* regulates transcriptional dosage via promoter methylation; however, an association with phenotype has not been reported (Druker et al., 2004). Together, these studies suggest that ERVs of the IAP subclass have the potential to be variably methylated, here referred to as variably methylated IAPs (VM-IAPs).

The properties and underlying mechanisms governing the establishment, behavior, and inheritance of VM-IAPs remain elusive, as does the extent to which they represent a genome-wide phenomenon. 45% of the murine genome is made up of repetitive sequences, with ERVs comprising about 12% of the genome. In the C57BL/6J genome, there are approximately 12,000 ERVs of the IAP subclass (Smit et al., 2015). The degree to which this substantial fraction of the repeat genome might modulate phenotype is unclear, and the total number of naturally existing murine VM-IAPs is unknown to date.

Previous studies have searched for metastable epialleles with limited success. Strategies have included surveying expression microarray data for within-strain interindividual expression patterns, screening for retrotransposons that neighbor promoters marked by the active histone modification H3K4me3, and conducting a phylogenetic analysis on IAP elements (Weinhouse et al., 2011, Ekram et al., 2012, Faulk et al., 2013). A recent more extensive screen used comparative whole-genome bisulfite sequencing (WGBS) data and described 55 ERV regions exhibiting some interindividual differential methylation, with validation in two tissues shown for four (Oey et al., 2015). This study confirmed that naturally occurring germline mutations and interindividual genetic differences do not underlie the epigenetic variation observed at the identified regions. While individually informative, there is little or no overlap between the results of these screens. The more challenging task of identifying human metastable epialleles has been tackled before, but the genetic heterogeneity associated with human cohorts remains a significant hindrance in such studies (Silver et al., 2015).

Here, we report a novel high-stringency genome-wide approach to comprehensively identify VM-IAPs. Using WGBS and RNA sequencing (RNA-seq) datasets generated from pure non-cycling populations of *ex vivo* purified naive B and T cells, we identified individual elements possessing features of metastable epialleles. After extensive validation, we have characterized their relationship to each other and to the vast majority of IAPs in the genome that are fully and stably modified. Furthermore, we determined their patterns of inheritance from one generation to the next. Our study identifies a repertoire of loci with the potential to act as markers of normal and compromised environmental contexts and as tools to uncover mechanisms of non-genetic inheritance and, more generally, to provide insights into the mechanisms of silencing at repeats and the impact of mammalian repetitive elements on genome function and phenotype.

## Results

### Identification of the VM-IAP Methylation Pattern

A three-step approach was used to identify VM-IAPs with metastable epiallele properties genome-wide. Our starting point for defining a metastable epiallele was (a) interindividual methylation variation at the IAP-LTR promoter, (b) consistent intraindividual methylation, and (c) variation in expression at an adjacent gene, as described for previously identified metastable epialleles. To this end, the first step utilized a catalog of polymorphic IAPs “private” to C57BL/6J compared to CAST/EiJ mice and screened for C57BL/6J-specific IAPs potentially impacting the expression of neighboring genes. The second step used the set of identified VM-IAP candidates from the first step to develop an algorithm to identify VM-IAPs genome-wide, which was applied to all IAPs in the C57BL/6J genome regardless of impact on adjacent expression. The third step consisted of running the algorithm on all C57BL/6J ERVs to assess the extent to which other ERV subclasses can act as metastable epialleles.

According to published data, 1994 IAP insertions are present in the C57BL/6J mouse strain and absent from the CAST/Eij strain (Nellåker et al., 2012). For the first step of our screen, we hypothesized that such polymorphic IAP insertions could explain some of the differential gene expression observed between the two strains. Differentially expressed genes were identified using RNA-seq datasets generated from naive non-cycling B and T cell populations purified from C57BL/6J and CAST/Eij mice generated as part of the BLUEPRINT reference epigenome project (Adams et al., 2012; accession number: GSE94676). The 552 polymorphic IAPs lying within or near differentially expressed genes were selected as potential metastable epialleles.

**Figure 1:**
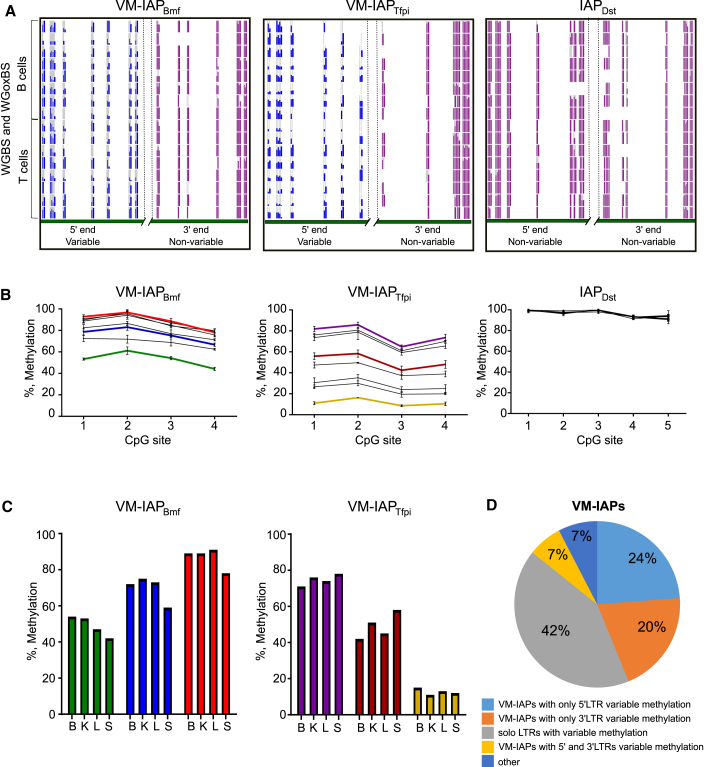
Identification of VM-IAPs (A) WGBS and whole-genome oxidative bisulfite sequencing (WGoxBS) tracks of the distal regions of the 5′ and 3′ LTRs belonging to VM-IAP_Tfpi_ (chr2:84505209-84510421), VM-IAP_Bmf_ (chr2:118554765-118558375), and control hypermethylated IAP_Dst_ (chr1:34347456-34351369). The poorly mapped central portion of the elements was removed due to its repetitive nature. Each vertical line represents one CpG, and each horizontal track represents one of 16 biological replicates. CpGs in variably methylated LTRs are highlighted in blue, illustrating ragged LTR methylation. CpGs in highly methylated LTRs are highlighted in purple. (B) Bisulphite pyrosequencing validation of interindividual methylation levels in C57BL/6J kidney tissues (n = 8) at VM-IAP_Tfpi_, VM-IAP_Bmf_, and IAP_Dst_. Each individual is represented by a single line. Sequenced CpGs are the most distal CpGs of the variably methylated LTRs. Colors are individual specific and correspond to those used in (C). (C) Intraindividual methylation consistency at VM-IAP_Tfpi_ and VM-IAP_Bmf_ across brain (“B”), kidney (“K”), liver (“L”), and spleen (“S”). Individual-specific colors correspond to those in (E). (D) Distribution of VM-IAPs according to methylation variation region. The number of VM-IAPs in each category is shown. See also Figures S1 and S2 and Table S1.

Methylation profiles of the identified 552 C57BL/6J IAPs were extracted from datasets generated from the same B and T cell populations used for the RNA-seq datasets. Both cell types were used because the methylation level at a metastable epiallele is established early in development and is therefore consistent within the same individual (Dolinoy et al., 2006, Waterland et al., 2006). As expected, the vast majority of these IAPs were highly methylated across all datasets. However, 31 showed a distinct methylation pattern at the IAP LTR characterized by “ragged” methylation levels between replicates (Figure 1A).

To test the hypothesis that ragged methylation reflected interindividual methylation variation, different tissues were isolated from 10 C57BL/6J mice and used to experimentally assess the methylation level of distal CpGs at the 5′ end of the candidate VM-IAPs using bisulfite pyrosequencing. IAPs with ragged methylation showed clear interindividual variation in methylation, while fully methylated IAPs did not (Figure 1B). Furthermore, these regions had consistent methylation levels across tissues within a single individual. Thus, within an individual animal, each VM-IAP exhibited its own level of methylation, and this level was evident in all somatic tissues of that individual, as described for *A*^*vy*^ and *Axin*^*Fu*^ (Figure 1C). These findings confirm that ragged methylation can represent interindividual methylation variation, providing a framework for the second stage of the screen: the unbiased genome-wide identification of VM-IAPs.

### Genome-wide Identification of VM-IAPs

**Figure S1:**
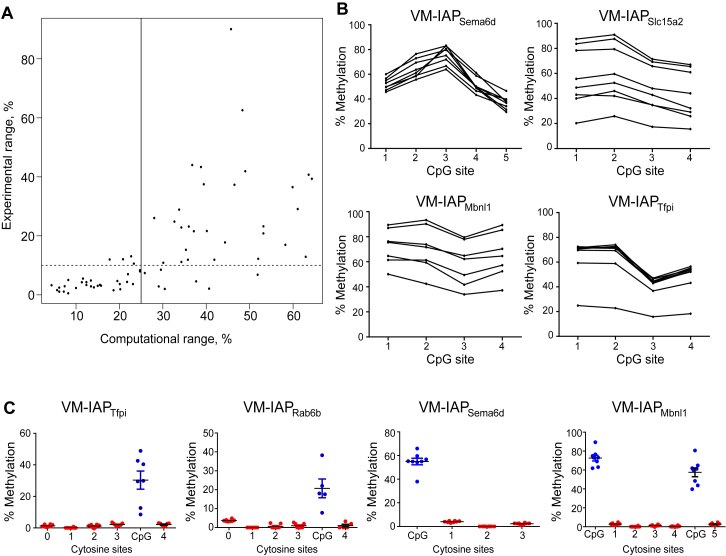
Validation and Characterization of VM-IAPs, Related to Figure 1 (A) Validation of methylation variation threshold used for the genome-wide screen. Each dot represents an IAP. Experimental range represents the difference between average methylation levels of the most highly and lowly methylated individuals, identified via bisulfite pyrosequencing. The computational and experimental ranges are correlated (two-tailed Pearson). The vertical line defines the threshold used for the genome-wide screen and the dotted horizontal line represents the upper range of experimental error associated with pyrosequencing. (B) Inter-individual methylation variation at VM-IAP_Tfpi_, VM-IAP_Mbnl1,_ VM-IAP_Slc15a2,_ and VM-IAP_Sema6d_, tested in pure B cell populations extracted from eight individuals, confirming the relationship between the BLUEPRINT experimental data generated from whole C57BL/6J tissues. (C) Non-CG methylation at four VM-IAPs, assessed by bisulfite pyrosequencing. Each dot represents an individual. Non-CG cytosines are shown as red dots and CpGs are shown as blue dots.

To assess the full extent of VM-IAPs in the C57BL/6J genome, the ragged methylation pattern observed for our initial set of VM-IAPs was used to generate a genome-wide algorithm. The algorithm generated a value reflecting the methylation variation of each IAP in the C57BL/6J genome and was independent of expression of adjacent sequences. Computational variations at 68 IAP-LTRs (ranging from 5% to 64%) were experimentally verified to determine the accuracy of the algorithm and establish a threshold for true methylation variation (Figure S1A and Table S1). 25% methylation variation between the second-highest and second-lowest average methylation level between biological replicates was selected as the threshold for further analysis, as greater than 75% of randomly selected IAPs within this range showed more than 10% interindividual methylation variation upon experimental validation. This approach resulted in the identification of around 100 candidate VM-IAPs (Table S2).

We confirmed that the observed *in vivo* interindividual methylation variation was not a technical artifact resulting from tissue-type heterogeneity by repeating bisulfite pyrosequencing on pure populations of B cells from different mice and successfully validating interindividual methylation variation (Figure S1B). Given the well-established role of non-CpG methylation in transposable element silencing in plants (Stroud et al., 2014), we assessed VM-IAPs for non-CpG methylation. None was detected (Figure S1C).

### VM-IAPs Are Evolutionarily Young Insertions with Locus-Specific Methylation States

**Figure S2:**
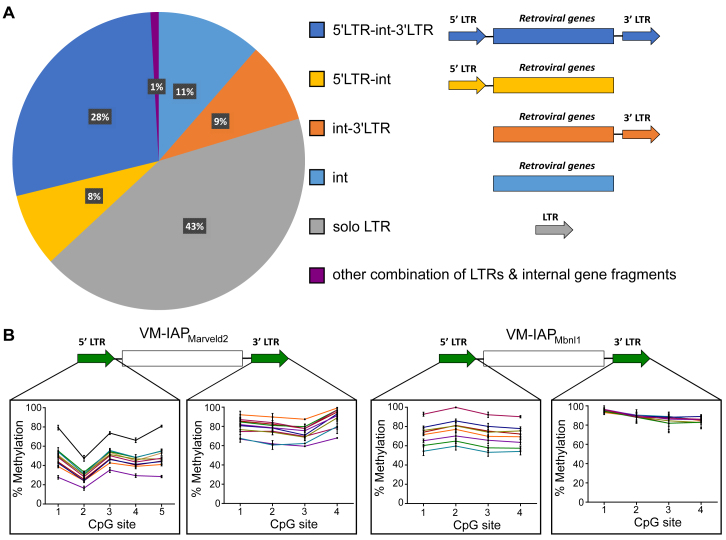
Structural Analysis of VM-IAPs, Related to Figure 1 (A) Pie chart distribution of all IAPs in the C57BL/6J genome based on IAP structure, where “int” refers to internal retroviral sequence. (B) Examples illustrating that LTRs behave independently of each other. Bisulphite pyrosequencing of 5′ and 3′ LTRs of VM-IAP_Marveld2_ and VM-IAP_Mbnl1_. The same individuals were used to assess both LTRs, color-coded accordingly.

A full-length intact IAP consists of 5′ and 3′ LTRs flanking retroviral genes (Falzon and Kuff, 1988, Mietz et al., 1987). Over evolutionary time, the structure of full-length IAPs is disrupted by deletions, point mutations, and recombination, eventually preventing retrotransposition activity (Stoye, 2001). The full range of IAP structures was represented in the identified set of candidate VM-IAPs, including full-length IAPs, truncated IAPs missing either their 5′ or 3′ LTR, solo LTRs, and truncated IAPs with no LTRs at all (Figure S2). Experimental validation of full-length VM-IAPs showed that 5′ LTRs differed from their corresponding 3′ LTRs in both their range and degree of methylation variation (Figures 1D and S2B). This indicates that 5′ and 3′ LTRs acquired their methylation variation independently of each other despite virtually identical genetic sequences.

**Figure 2:**
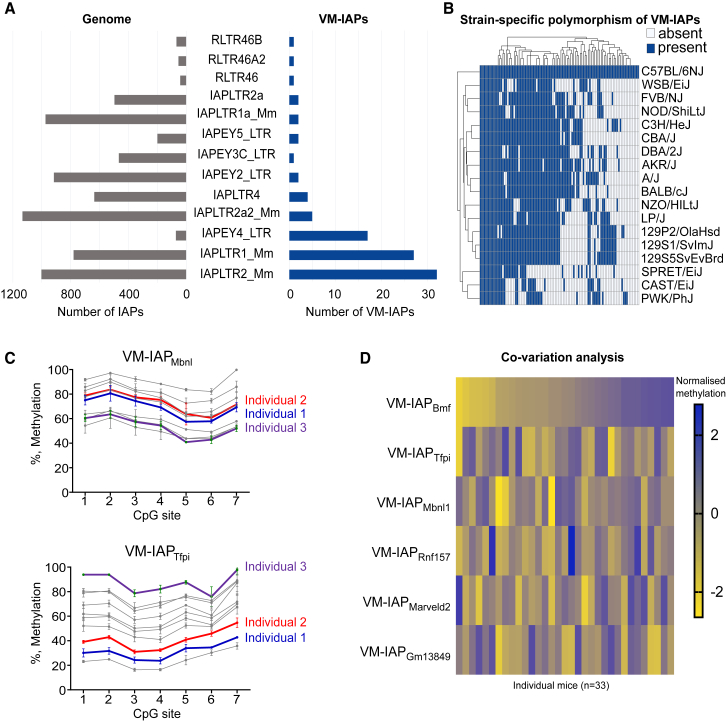
VM-IAPs Are Evolutionarily Young Insertions with Locus-Specific Methylation States (A) Enrichment of IAP subtypes in VM-IAPs. The left side in gray represents the total number of IAPs in the genome of a particular LTR subtype. The right side in navy shows the number of VM-IAPs of a particular LTR subtype. (B) Heatmap showing the presence or absence of VM-IAPs across 18 mouse strains, as determined from the Mouse Genomes Project (https://www.sanger.ac.uk/science/data/mouse-genomes-project). VM-IAPs are clustered by strain and presence of the IAP relative to C57BL/6J. (C) Bisulphite pyrosequencing of VM-IAP_Tfpi_ and VM-IAP_Mbnl1_ in the same eight C57BL/6J mice. VM-IAP_Tfpi_ and VM-IAP_Mbnl1_ have identical sequences yet are differentially methylated within the same individual. Sequenced CpGs are the seven most distal ones of the 5′ LTRs. Specific individuals are color coded. (D) Methylation levels at six VM-IAPs in 33 C57BL/6J mice are compared in a co-variation analysis. Methylation levels were normalized to a given VM-IAP’s interindividual methylation range. Data show no relationship between VM-IAP methylation states within an individual. See also Figure S3.

The IAP nomenclature system used by the University of California, Santa Cruz (UCSC) Genome Browser classifies IAPs based on their LTR structural differences. This classification was used to investigate subtype enrichment of VM-IAPs. The majority of VM-IAPs had either IAPLTR1_Mm or IAPLTR2_Mm flanking LTRs (Figure 2A). Interestingly, IAPLTR1_Mm LTRs belonged to VM-IAPs with viral gene sequences while IAPLTR2_Mm elements were mainly solo LTR VM-IAPs. The IAPLTR1_Mm IAP subtype is considered to be the evolutionarily youngest IAP subtype (Qin et al., 2010). Furthermore, using data from the Mouse Genomes Project, we analyzed the presence or absence of VM-IAPs across 18 mouse strains and found that the majority of VM-IAPs were polymorphic insertions (Figure 2B).

**Figure S3:**
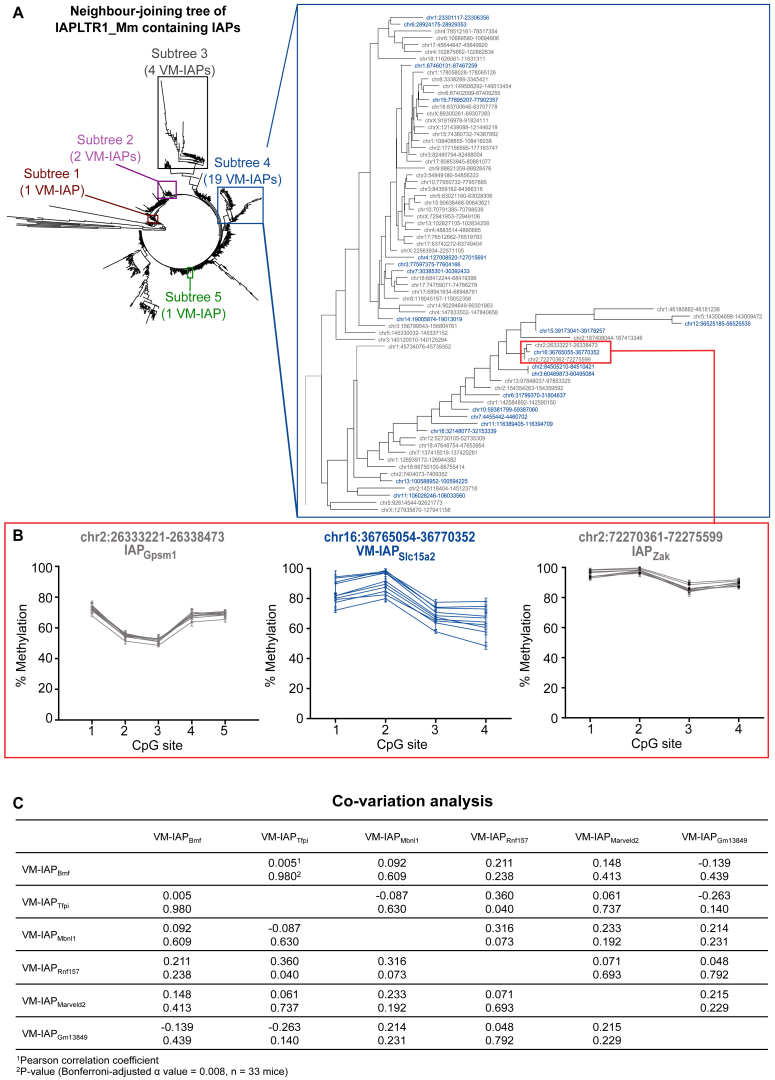
Genetic Sequence and Co-variation Analyses of VM-IAPs, Related to Figure 2 (A) Neighbor-joining tree for IAPLTR1_Mm elements, made using Geneious software. VM-IAPs are distributed across 5 subtrees, shown in boxes. Subtree 4, containing the most VM-IAPs, is shown in more detail, with VM-IAP coordinates highlighted in blue. (B) IAP_Zak_ and IAP_Gpsm1_, closely related to VM-IAP_Slc15a2_ by sequence, do not exhibit inter-individual methylation variation upon bisulphite pyrosequencing (n = 10). (C) Normalized correlation matrix of methylation levels across 6 VM-IAPs in 33 mice.

Given the wide range of IAP structures and LTR subtypes, we investigated the possibility that the methylation state of VM-IAPs was exclusively determined by their genetic sequence. The IAPLTR1_Mm subtype was used to build a neighbor-joining tree using the sequences of all 780 IAPs of this LTR subtype in the C57BL/6J genome, including 27 VM-IAPs. Neighbor-joining tree analysis assesses sequence similarity and groups closely related IAPs together. Five distinct subtrees were identified containing VM-IAPs, with subtree 4 containing most of them (Figure S3A). This enrichment likely reflects their recent integration into the C57BL/6J genome and indicates that genetic sequence is at least partially involved in conferring methylation variation. Interestingly, VM-IAP_Slc15a2_, IAP_Gpsm1_ and IAP_Zak_ are highly clustered, but only VM-IAP_Slc15a2_ is a metastable epiallele, indicating that other factors, such as spatial organization, are likely at play (Figure S3B). We confirmed that sequence is not the sole determining factor for methylation level by comparing two VM-IAPs with 100% identical genetic sequences, showing they have different methylation levels within a given individual (Figure 2C).

We further explored co-variation of VM-IAP methylation levels within an individual by assessing the methylation state of six VM-IAPs in 33 different mice (Figure 2D). A normalized correlation matrix showed that the methylation level of each VM-IAP did not significantly correlate with that of other VM-IAPs within the same individual (Figure S3C). This indicates that the mechanism governing variable methylation likely acts in *cis* and argues against an overarching *trans-*mediated mechanism targeting all VM-IAPs within an individual in the same way.

### VM-IAPs are Flanked by CTCF Binding Sites

Our results showed that other factors in addition to recent integration are involved in driving the methylation pattern observed at candidate VM-IAPs. We therefore asked whether the genomic location of VM-IAP insertion sites sets them apart from other non-variable IAPs. VM-IAPs are randomly distributed in the genome and do not cluster in specific topologically associating domains (TADs), nor are they enriched at TAD boundaries (data not shown). We found that approximately 70% of VM-IAPs are intergenic, and only two of them fall in UTRs. The remaining are intronic (Table S2A).

**Figure S4:**
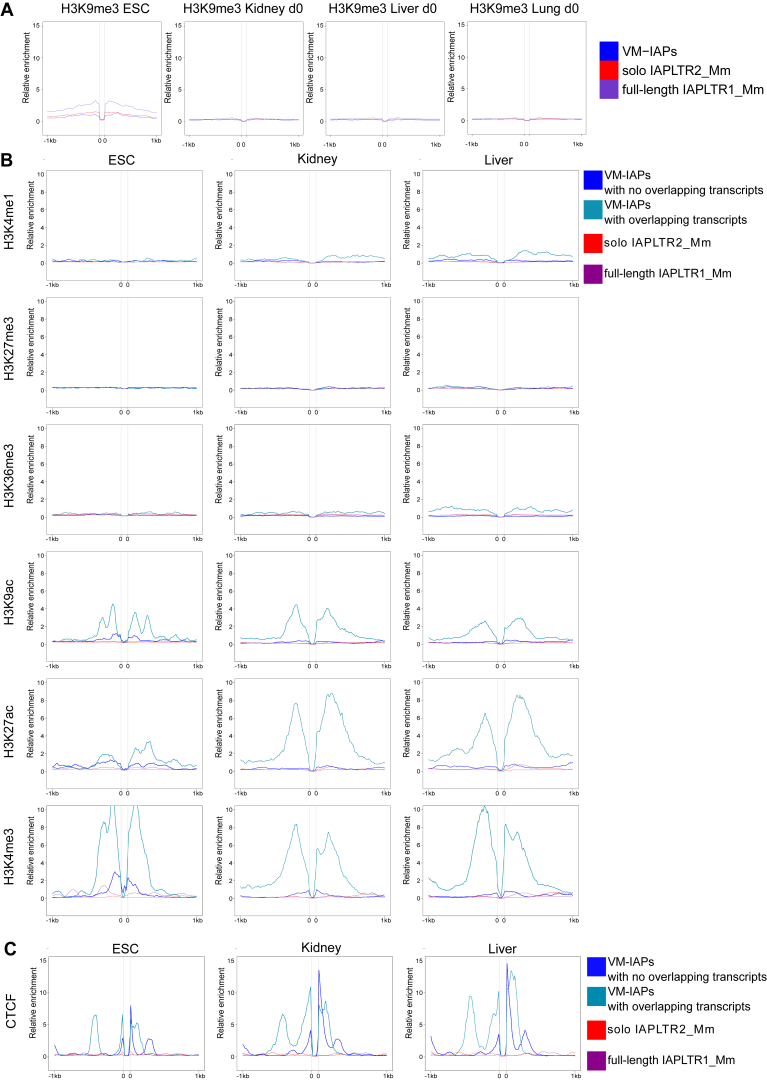
Epigenetic Profiles of VM-IAP Flanking Regions, Related to Figure 3 and 4 (A) Relative H3K9me3 enrichment profiles of VM-IAP flanking genomic regions in ESCs, kidney, liver and lung. VM-IAPs are not flanked by H3K9me3-enriched regions and are equivalent to full-length IAPLTR1_Mm and solo IAPLTR2_Mm IAPs. All IAPs were fit to 100bp, shown as the space between the two 0’s. 0’s represent the start and end coordinates of IAPs. ChIP-seq datasets were downloaded from ENCODE. (B) Epigenetic profiles of regions flanking VM-IAPs, separated by presence or absence of overlapping *de novo* assembled transcripts. Highly methylated full length IAPs of the IAPLTR1_Mm subclass and solo LTRs of the IAPLTR2_Mm subclass serve as controls. All IAPs were fit to 100bp, shown as the space between the two 0’s. 0’s represent the start and end coordinates of the IAPs. (C) Relative CTCF enrichment profiles of regions flanking VM-IAPs, separated by presence or absence of overlapping *de novo* assembled transcripts.

**Figure 3:**
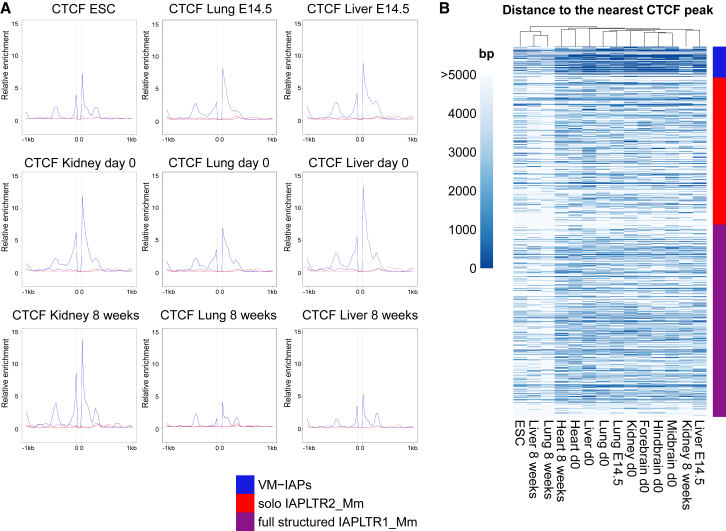
VM-IAPs Are Flanked by CTCF Binding Sites (A) Relative CTCF enrichment profiles of VM-IAP flanking genomic regions in C57BL6/J ESCs, kidney, lung, and liver. Developmental stages E14.5, day 0, and 8 weeks are shown. Highly methylated full-length IAPs of the IAPLTR1_Mm subclass and solo LTRs of the IAPLTR2_Mm subclass serve as controls. All IAPs were fit to 100 bp, shown as the space between the two zeros. Zeros represent the start and end coordinates of IAPs. ChIP-seq datasets were downloaded from ENCODE (ENCODE Project Consortium, 2012). (B) Heatmap of the distance from IAP border to the nearest CTCF peak across ChIP-seq ENCODE datasets. VM-IAPs, full structure IAPs of the IAPLTR1_Mm subclass, and solo LTRs of the IAPLTR2_Mm subclass are clustered and used in comparison. See also Figure S4.

We next analyzed ENCODE chromatin immunoprecipitation (ChIP)-seq datasets to explore the epigenetic profiles of regions flanking VM-IAPs (ENCODE Project Consortium, 2012). It has been shown in embryonic stem cells (ESCs) that strain specific polymorphic IAP insertions are capable of spreading heterochromatic marks to flanking genomic DNA (Rebollo et al., 2011). However, no clear difference in H3K9me3 distribution was found between regions flanking VM-IAPs and non-variable IAPs (Figure S4A). Strikingly, the majority of VM-IAPs are bordered by CCCTC binding factor (CTCF) binding (Figures 3A). This enrichment was observed in datasets generated from different somatic tissues and from different developmental time points, suggesting stable maintenance of CTCF binding near VM-IAPs throughout development. To further investigate this enrichment, we produced a heatmap of the distance from the IAP border to the nearest CTCF peak across 14 ENCODE ChIP-seq datasets and found that CTCF was closer to VM-IAPs than non-variable IAPs across all datasets (Figure 3B). CTCF is a methylation-sensitive DNA binding protein that is crucial for both preimplantation and postimplantation stages (Phillips and Corces, 2009). CTCF-deficient oocytes cannot progress to the blastocyst stage following fertilization, and CTCF knockout embryos die before implantation (Wan et al., 2008, Moore et al., 2012). This developmental time point is in line with the proposed time point for the establishment of methylation at *A*^*vy*^ (Waterland et al., 2006, Blewitt et al., 2006). CTCF prefers unmethylated binding sites and has been shown to inhibit Dnmt1 activity to prevent methylation of its binding domain (Bell and Felsenfeld, 2000, Zampieri et al., 2012). It is an intriguing possibility that an interplay between IAP methylation and CTCF binding-site hypomethylation is involved in the establishment and/or maintenance of VM-IAPs.

### VM-IAPs Can Interfere with Transcriptional Events

**Figure 4:**
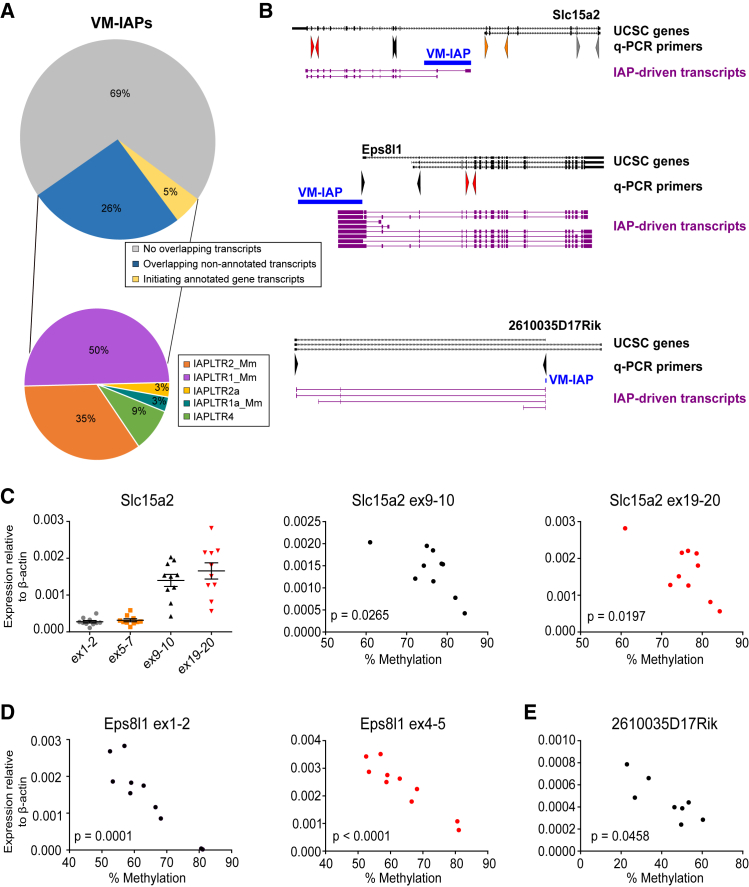
VM-IAPs Can Interfere with Tissue-Specific Transcriptional Events (A) Pie charts of percentage of VM-IAPs overlapping *de novo* assembled transcripts and their distribution according to IAP-LTR subtype. (B) VM-IAP-associated ectopic transcription of *Slc15a2*, *Eps8l1*, and *2610035D17Rik*. Intragenic VM-IAP_Slc15a2_ drives expression of downstream *Slc15a2* exons. Intergenic VM-IAP_Eps8l1_ drives ectopic expression of *Eps8l1*. Intragenic VM-IAP_2610035D17Rik_ provides an alternative promoter for lincRNA *2610035D17Rik*. VM-IAPs are shown in blue and *de novo* assembled transcripts in purple. Transcripts extracted from UCSC are in black, and qPCR primers are depicted as arrows and color coded to correspond with (C), (D), and (E). (C) Expression of *Slc15a2* downstream exons 9–10 and 19–20 (spleen) is inversely correlated with VM-IAP_Slc15a2_ methylation (two-tailed Pearson). Upstream exons are not expressed. Expression was quantified by qPCR and shown relative to housekeeping gene *β-actin*. Each dot represents a different individual. (D) Expression of *Eps8l1* exons 1–2 and 4–5 (brain) is inversely correlated with VM-IAP_Eps8l1_ methylation (two-tailed Pearson). (E) Expression of the VM-IAP_2610035D17Rik_-driven transcript (spleen) is inversely correlated with VM-IAP_2610035D17Rik_ methylation (two-tailed Pearson). See also Figure S4.

The methylation states of the IAPs integrated at the *A*^*vy*^ and *Axin*^*Fu*^ loci have been shown to have a direct impact on adjacent gene transcription and the phenotype of individual animals. In these cases, transcription initiates at the IAP LTR promoter, creating a chimeric transcript extending through the adjacent gene (Duhl et al., 1994, Vasicek et al., 1997). To explore the extent to which VM-IAPs can initiate transcription of endogenous genes, we analyzed *de novo* transcriptome assemblies generated from pure non-cycling populations of B and T cells (GEO: GSE94676). Only five VM-IAPs appeared to initiate transcripts overlapping annotated genes (Figure 4A). We investigated whether the expression level of these transcripts was related to the methylation level of the VM-IAPs and found statistically significant inverse correlations between gene expression and VM-IAP methylation levels, as observed for *A*^*vy*^ and *Axin*^*Fu*^ (Figures 4B–4D). This finding reinforces the idea that metastable epialleles are capable of driving gene expression, yet reveals that promoter activity at these regions is an exception rather than the rule.

We next queried the number of VM-IAPs overlapping with transcripts in general and identified less than one-third of VM-IAPs with this property. Their insertion sites were enriched for the active histone marks H3K27ac, H3K9ac, and H3K4me3 (Figure S4B). No clear enrichment for H3K36me3 was observed (Figure S4B). It is therefore probable that the association of VM-IAPs with these transcripts is a product of insertion into transcriptionally active or “open” genomic regions. We found that half of the transcript-initiating or transcript-overlapping VM-IAPs were full-length IAPs with IAPLTR1_Mm LTRs, suggesting that evolutionarily young VM-IAPs are more likely to have a transcriptional influence (Figure 4A). Of note, CTCF binding at VM-IAP borders was independent of the presence or absence of overlapping transcripts (Figure S4C), suggesting a potential functional role involving long-range interactions.

### Methylation Variability Is Lost in the Male Germline and Re-established in the Next Generation

**Figure 5:**
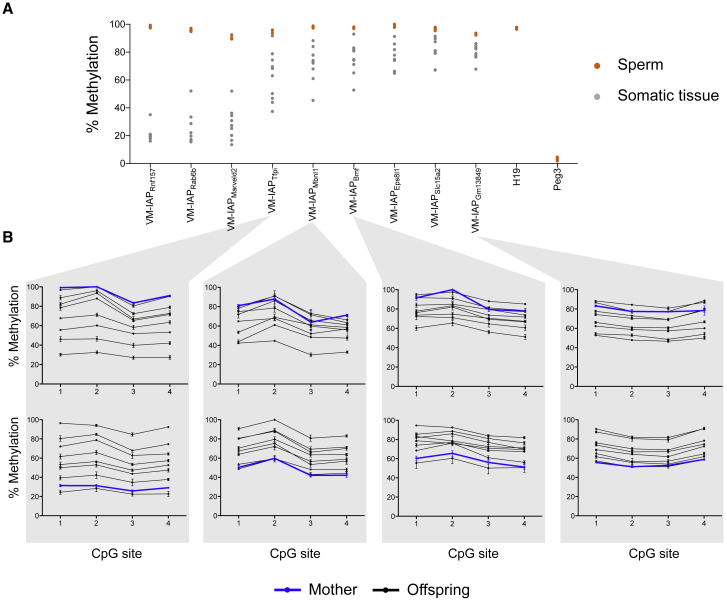
VM-IAPs Are Hypermethylated in the Male Germline and Reconstructed as Variable Loci in the Next Generation (A) VM-IAPs are hypermethylated in sperm. Methylation levels at nine VM-IAPs in C57BL/6J sperm and corresponding somatic tissue are shown (n = 8–10 mice). Paternally expressed *Peg3* and maternally expressed *H19* serve as germ cell purity controls. Values shown are averages across the methylation level of the four distal CpG sites of the VM-IAP 5′ LTRs assessed by bisulphite pyrosequencing. (B) Highly and lowly methylated mothers produce the full range of variably methylated offspring (n = 8 pups). Offspring and maternal methylation levels at VM-IAP_Tfpi_, VM-IAP_Mbnl1_, VM-IAP_Bmf_, and VM-IAP_Gm13849_ were assessed from ear samples by bisulphite pyrosequencing. See also Figure S5.

To evaluate heritability dynamics at VM-IAPs, we first assessed their methylation state in sperm. Nine VM-IAPs with wide methylation variation ranges were analyzed for LTR methylation levels in both somatic and mature sperm isolated from adult C57BL/6J male mice. As expected based on experimental validation, VM-IAPs showed interindividual methylation variation in the male somatic samples. In contrast, all VM-IAPs analyzed were fully methylated in sperm for all individuals, and no interindividual variation was observed (Figure 5A). Although repeat elements tend to be heavily methylated in sperm (Kobayashi et al., 2012), this is inconsistent with previous observations at *A*^*vy*^, where partial methylation in sperm was observed to reflect the methylation status in somatic tissues despite the absence of heritability of phenotype from the sire (Rakyan et al., 2003).

We next sought to determine whether the methylation state of VM-IAPs is variable from one generation to the next. For the four VM-IAPs analyzed, the somatic methylation levels of pups born to highly or lowly methylated C57BL/6J mothers showed the full range of methylation variation observed in the previous generation (Figure 5B). This demonstrates that the methylation variability of VM-IAPs is faithfully reconstructed in the F1 generation after passage through the male and female germlines and that the methylation level of an individual does not influence its ability to produce offspring with the full methylation range associated with that VM-IAP.

**Figure S5:**
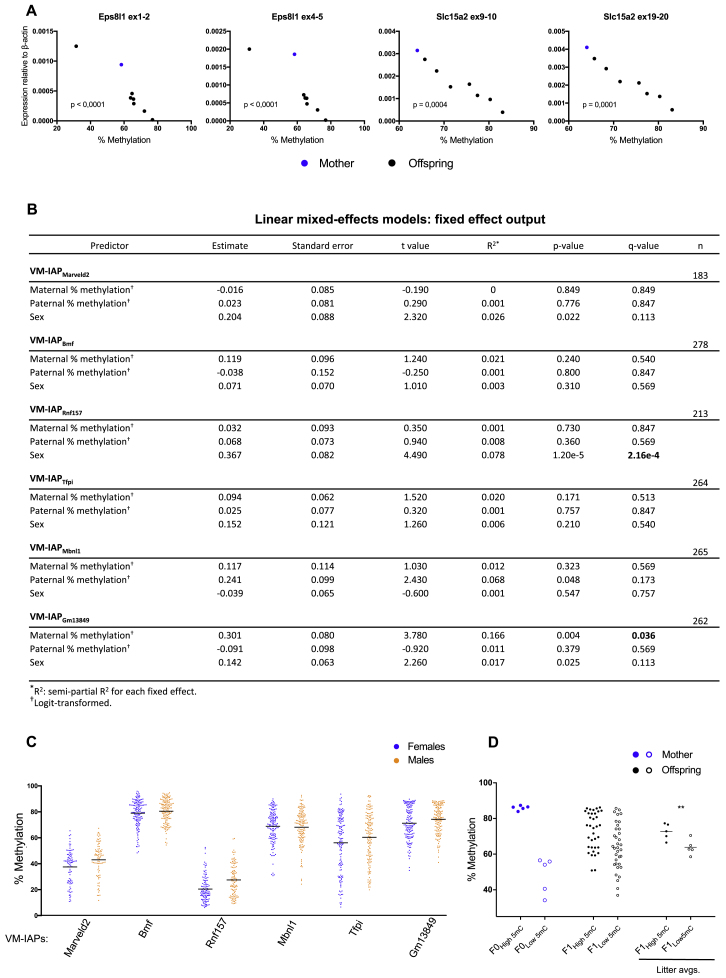
Transcriptional and Statistical Results of VM-IAP Inheritance Studies, Related to Figures 5 and 6 (A) The inverse correlation between expression of *Eps8l1* (exons 1-2 and 4-5) and VM-IAP_Eps8l1_ methylation is recapitulated in the F1 generation (two-tailed Pearson). This is also observed for expression of *Slc15a2* (exons 9-10 and 19-20) and VM-IAP_Slc15a2_ methylation. Expression was quantified in spleen by qPCR and shown relative to housekeeping gene *β-actin*. Each dot represents a different individual and maternal expression is shown in blue. (B) Linear mixed-effects models (LMMs) of offspring methylation for six VM-IAPs using the lmerTest package in R. Maternal methylation level, paternal methylation level, and sex were treated as fixed effects. Breeding pair and litter were treated as random effects. Output for the fixed effects is presented. Raw p values were generated using the Satterthwaite approximation for degrees of freedom. Adjusted p values (q-values) were generated using the Benjamini & Hochberg correction to account for multiple testing. (C) Methylation levels of offspring used to fit LMMs, separated by sex. A significant sex effect is observed for VM-IAP_Rnf157_ (p value: 1.20e-5; q-value: 2.16e-4). Average methylation levels are shown in black bars. (D) Validation of the observed maternal effect on offspring methylation levels at VM-IAP_Gm13849_. The average methylation levels of the first litters born to five highly methylated and five lowly methylated C57BL/6J females were assessed from ear notches via bisulfite pyrosequencing (one-sided t test; p value: 0.0069; n = 5 litters per group).

To determine whether the inverse correlation between VM-IAP methylation and adjacent gene expression observed for a subset of VM-IAPs endures in the next generation, we assessed expression and methylation levels of VM-IAP_Eps8l1_ and VM-IAP_Slc15a2_ in maternal and offspring spleen tissues. We found that expression levels were variable among F1 littermates and inversely correlated with VM-IAP methylation levels (Figure S5A). Together, these findings indicate that the unique methylation signature of each VM-IAP and its effect on transcription is re-established transgenerationally.

**Figure 6:**
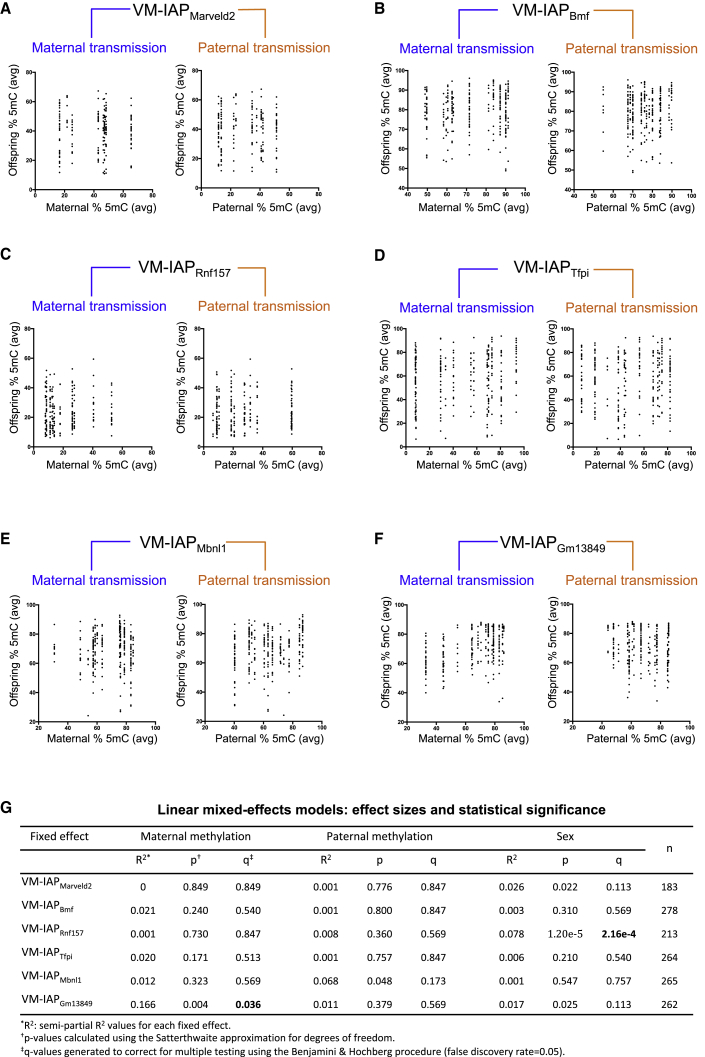
Inheritance Analysis of VM-IAPs (A–F) Methylation levels at VM-IAPs were quantified from ear samples taken from C57BL/6J breeding pairs and their offspring. Offspring methylation level is plotted against maternal and paternal methylation level for VM-IAP_Marveld2_ (A), VM-IAP_Bmf_ (B), VM-IAP_Rnf157_ (C), VM-IAP_Tfpi_ (D), VM-IAP_Mbnl1_ (E), and VM-IAP_Gm13849_ (F). Methylation levels represent averages across the first four distal CpG sites of the VM-IAP 5′ LTRs. (G) Statistical output from LMMs showing a significant maternal effect on offspring methylation at VM-IAP_Gm13849_ as well as a significant sex effect on VM-IAP_Rnf157_ methylation levels. Analyses were carried out using the lmer() function from the lme4 R package with maternal methylation level, paternal methylation level, and sex treated as fixed effects. Breeding pair and litter were treated as random effects. Raw p values were generated using the Satterthwaite approximation for degrees of freedom. Adjusted p values (q) were generated using the Benjamini and Hochberg correction to account for multiple testing. Semi-partial R^2^ values, representing the effect size for each fixed effect, were calculated using the r2beta() function from the r2glmm R package. Sample sizes are shown. See also Figure S5.

The *A*^*vy*^ locus is widely studied in large part because it exhibits epigenetic inheritance. In a C57BL/6J genetic background, the phenotype of the dam, but not the sire, influences the phenotypic distribution observed in the offspring (Morgan et al., 1999). In breeding-intense experiments, we investigated whether this was the case for six novel VM-IAPs. DNA methylation levels at the IAP LTR promoter were quantified in adult C57BL/6J breeding pairs and their offspring (Figures 6A–6F). We asked whether parental methylation level affected offspring methylation by building linear mixed-effects models (LMMs). Using LMMs allowed us to incorporate breeding pairs and litters as random effects, thereby controlling for the non-independence of siblings and littermates, respectively. We included maternal methylation level, paternal methylation level, and sex as potentially predictive fixed effects (Figure S5B).

For five out of the six regions tested, neither maternal nor paternal methylation level had a significant effect on offspring methylation levels (Figure 6G). For VM-IAP_Gm13849_, the maternal methylation level, but not the paternal one, significantly affected offspring methylation levels (p = 0.004; q = 0.036; Figures 6G and S5B). This difference in heritability between parental lineages is consistent with the pattern observed for *A*^*vy*^ on a C57BL/6J background. Of note, while the maternal effect at this locus was significant, the R^*2*^ was small, indicating that maternal methylation only accounts for a small fraction of the methylation variability observed in the next generation (R^2^ = 0.166). Interestingly, when assessing whether the sex of an individual contributes to its methylation level, we found a highly significant sex effect at VM-IAP_Rnf157_ whereby males are more likely to exhibit higher methylation levels than females (p = 1.20e−5; q = 2.16e−4; Figures 6G, S5B, and S5C). Weak evidence for a similar trend was observed for VM-IAP_Marveld2_ and VM-IAP_Gm13849_. This suggests there may be sex-linked modifiers of VM-IAPs.

Given that a single VM-IAP showed maternal heritability, we designed a smaller-scale experiment on VM-IAP_Gm13849_ to validate this finding in a separate set of mice. We selected five highly methylated and five lowly methylated C57BL/6J females for breeding and subsequently assessed the VM-IAP_Gm13849_ methylation levels of offspring from their first litter. We found that the methylation level of offspring born to highly methylated mothers was significantly different from that of offspring born to lowly methylated mothers, validating our previous result (p = 0.0069; n = 5 litters per group; Figure S5D). Together, our heritability studies indicate that inheritance of methylation levels is not a universal feature of VM-IAPs and instead show the remarkable reprogramming and faithful re-establishment of VM-IAP variable states from one generation to the next regardless of parental methylation level.

### Non-IAP ERVs Can Exhibit Interindividual Methylation Variation

The same genome-wide screening strategy used to identify VM-IAPs was implemented to analyze other types of ERVs. Since the algorithm was based solely on the magnitude of methylation variation, it does not distinguish different ERV classes. 208 ERV1, 760 ERVK, and 174 ERVL candidates were identified as potential variably methylated ERVs (VM-ERVs) using the threshold levels developed in the initial model. 44 ERVs were randomly selected for experimental validation via bisulfite pyrosequencing. 13 of them validated as true VM-ERVs, showing more than 10% interindividual methylation variation (Table S3). Hence, although our WGBS-based screen identified a plethora of ERVs as VM-ERV candidates, less than 30% were true positives. This level of false positives might reflect the challenges of repetitive element alignment and/or differences in the CpG density at the LTR of different ERV subclasses influencing the application of the algorithm. Experimental validation and comparative analysis of these and the set of candidate VM-IAPs identified in this study will ascertain the full extent of epigenetic metastability at all LTR retrotransposons across the genome.

**Figure S6:**
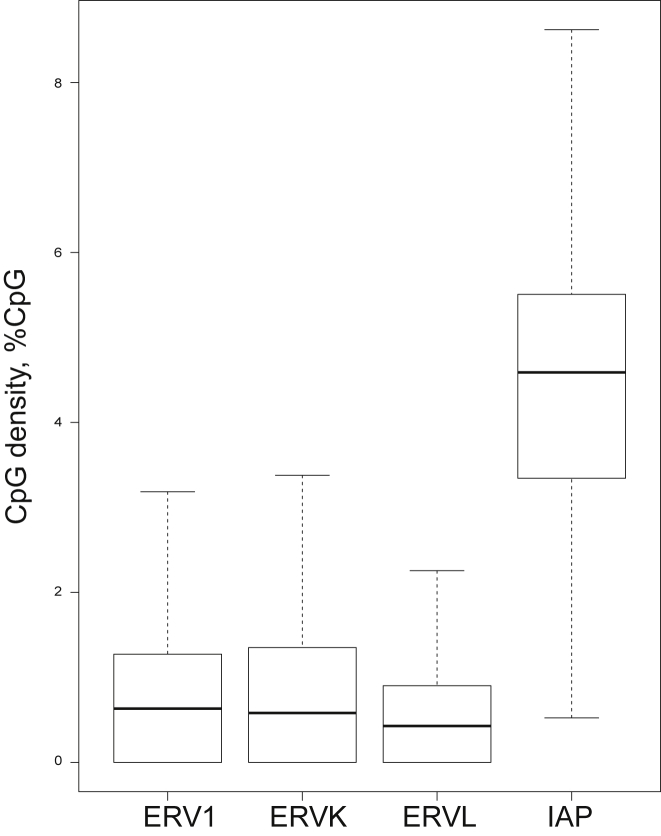
Classification of ERV Subtypes by CpG Density, Related to STAR Methods ERV LTR CpG density, separated by subtype. DNA sequences for all ERV LTR regions were extracted and sorted according to RepeatMasker annotation. CpG density was calculated as the percentage of CpGs to base pair length of the LTR region.

We computed the CpG density of different ERV-LTR subtypes in the genome and found that IAP LTRs indeed have the highest CpG density across all ERVs (Figure S6). It is therefore possible that CpG density might play a role in the properties of a metastable epiallele. This is in line with previous findings highlighting the particular susceptibility of IAPs to methylation-mediated regulation (Walsh et al., 1998).

## Discussion

A genome-wide screen identified multiple C57BL/6J VM-IAPs. Three of them (VM-IAP_Eps8l1_, VM-IAP_Bmf_, and VM-IAP_2610035D17Rik_) had previously been noted in a screen for the presence of H3K4me3 at retroelement promoters (Ekram et al., 2012). No H3K4me3 enrichment was found at lowly methylated *A*^*vy*^ individuals (Dolinoy et al., 2010)—this is consistent with our findings indicating a lack of H3K4me3 at VM-IAPs. A previous screen for metastable epialleles, encompassing all genomic regions but including only limited follow-up, identified 51 variably methylated ERV candidates. 22 overlapped with VM-IAPs identified in our study; the others were not detected in our screen (Oey et al., 2015). The previously identified metastable epiallele IAP_Cdk5rap1_ (Druker et al., 2004) fell just below the stringent threshold of 25% used here. For this reason, in addition to the list of novel VM-IAPs, we have provided an additional set of IAPs picked up in our screen using the methylation variation observed at IAP_Cdk5rap1_ as a threshold (Table S2, bottom section). However, this additional set contains several experimentally identified false positives and exhibits much narrower interindividual methylation ranges compared to the candidate VM-IAPs identified in our analysis.

Applying our screening strategy to other types of ERVs resulted in high false-positive rates upon experimental validation. This highlights the importance of rigorous experimental testing of candidates identified in studies on repeat elements and calls into question previous studies identifying non-IAP-derived metastable epialleles without conducting extensive validation. It remains challenging to develop bioinformatic approaches that distinguish epigenetically variable regions from poorly mapped ones. We nonetheless show that non-IAP ERVs are capable of exhibiting interindividual methylation variation and propose that the intriguing enrichment in IAP elements might reflect a requirement for high CpG density for the establishment of interindividual methylation variation.

We did not identify any specific genetic sequence features that explain acquisition of interindividual methylation variation at IAPs. In fact, very closely related VM-IAPs were found to have quite different ranges of methylation variation. In some cases, near-perfect sequence identity was observed between a VM-IAP and a non-variable IAP. However, we did find an enrichment for young classes of IAPs (IAPLTR1_Mm and IAPLTR2_Mm). It is therefore possible that VM-IAPs represent evolutionarily young IAPs in the process of becoming epigenetically silenced. In addition, VM-IAPs show an enrichment for strain-specific polymorphic IAPs, suggesting that recent integration into the C57BL/6J genome may contribute to their variably methylated states. Further analysis of the relationship between the acquisition of variable methylation and the strain-specific placement of such retrotransposons will contribute to our understanding of the mechanism through which they escape the fate of their fully methylated counterparts.

Unlike *A*^*vy*^, the majority of VM-IAPs described in this study do not initiate transcription events, indicating that these elements are not generally functioning as heterologous promoters. We found, however, that transcript expression that is initiated at VM-IAPs is low, and we detected no ontology enrichment for those genes adjacent to VM-IAPs.

Most compelling, however, is the finding that VM-IAPs are enriched for CTCF binding in their flanking regions. Since CTCF is a methylation-sensitive DNA binding protein preferring unmethylated DNA (Phillips and Corces, 2009), this association suggests a functional antagonism between methylation of an IAP and the maintenance of an unmethylated state at the CTCF binding site, leading to the early acquisition of a stochastically methylated state. Extensive further experiments will be required to decipher the potential relationship between CTCF binding and VM-IAPs. It has been proposed that VM-IAPs may have a selective advantage, conferring stochastic fitness and enhanced evolution (Branciamore et al., 2014, Branciamore et al., 2015). Taking into consideration a potential role for VM-IAPs in influencing long-range *cis*-acting interactions and the interstrain differences in the absence or presence of some of these elements, this idea can now be tested experimentally through comparative functional analyses between individuals and between different genetic backgrounds.

The hypermethylation observed in sperm at VM-IAPs, reconstructed into a variably methylated state after fertilization, suggests reprogramming upon paternal inheritance. Given that the methylation state of the *A*^*vy*^ and *Axin*^*Fu*^ alleles in sperm has been reported to reflect the methylation state in somatic tissues of the same individual (Rakyan et al., 2003, Blewitt et al., 2006), one might have expected the VM-IAPs identified in this study to behave in a similar manner. It is possible, however, that *A*^*vy*^ and *Axin*^*Fu*^ alleles are unusual compared to other metastable epialleles. Alternatively, this inconsistency may reflect the fact that they arose as insertional mutations in non-C57BL/6J mouse strains but have been maintained on a C57BL/6J background for the experiments in question. Hence, these loci, being the only non-C57BL/6J segments of DNA in an otherwise C57BL/6J genome, may be more refractory to the C57BL/6J sperm methylation machinery. Alternatively, the difference may be technical, reflecting the different methods used to quantify methylation. Despite the acquisition of complete methylation occurring in the male germline, we show that VM-IAPs are re-established as variable loci in the next generation, indicating faithful reconstruction of their variable state from one generation to the next.

Only one of the VM-IAPs we examined showed evidence of a maternal methylation-level memory reminiscent of previously described *A*^*vy*^ inheritance dynamics in a C57BL/6J genetic background. For this lone VM-IAP, the highly quantitative nature of the methylation analysis indicated a small effect size. Our findings raise questions about the generalizability of non-genetic inheritance at metastable epialleles and suggest that variable methylation can be reprogrammed and reconstructed across generations in the absence of a memory of parental state by a process that may depend on the genetic context of the variably modified locus.

Importantly, our inheritance analysis highlights how consistent the range of interindividual methylation variation is at each VM-IAP regardless of parental methylation state. The mechanism by which the variable state of these unique elements is reprogrammed and precisely re-established in the next generation remains to be elucidated. Interestingly, the bordering regions of previously identified putative human metastable epialleles are enriched for ERVs and long interspersed nuclear elements (LINEs), suggesting that the link between repeat elements and epigenetic metastability may be conserved (Silver et al., 2015). The findings reported here establish a repertoire of murine loci to study mechanisms of non-genetic inheritance, the influence of the repeat genome on phenotype, and the epigenetic impact of normal and compromised environmental contexts.

## STAR★Methods

### Key Resources Table

**Table:** 

REAGENT or RESOURCE	SOURCE	IDENTIFIER
**Critical Commercial Assays**		

B Cell Isolation Kit	Miltenyi Biotec	Cat#130-090-862
AllPrep DNA/RNA Mini Kit	QIAGEN	Cat#80204
RNase-Free DNase Set	QIAGEN	Cat#79254
PyroMark Gold Q96 Reagents	QIAGEN	Cat#972804
Imprint DNA Modification Kit	Sigma	Cat#MOD50
RevertAid H Minus First Strand cDNA Synthesis Kit	Thermo Fisher Scientific	Cat#K1631
LightCycler 480 SYBR Green I Master	Roche	Cat#04707516001
HotStarTaq DNA Polymerase	QIAGEN	Cat#203203

**Deposited Data**		

WGBS, WGoxBS, and RNA-seq datasets	This paper	GEO: GSE94676

**Experimental Models: Organisms/Strains**		

Mouse: C57BL/6J	The Jackson Laboratory	JAX: 000664

**Oligonucleotides**		

qPCR primers, see Table S4	This paper	N/A
PCR and pyrosequencing primers, see Table S5	This paper	N/A

**Software and Algorithms**		

Prism 7 for Mac OS X	GraphPad Software	https://www.graphpad.com/scientific-software/prism/
Geneious 9.0.5	Kearse et al., 2012	https://www.geneious.com/
PyroMark Assay Design SW 2.0	QIAGEN	Cat#9019077
Primer3	Untergasser et al., 2012	http://primer3.ut.ee/
liftOver	Hinrichs et al., 2006	http://genome.ucsc.edu/
BEDTools 2.25.0	Quinlan and Hall, 2010	https://bedtools.readthedocs.io/en/latest/index.html
DESeq2 1.3.52	Love et al., 2014	https://bioconductor.org/packages/release/bioc/html/DESeq2.html
edgeR 3.5.27	Robinson et al., 2010	https://bioconductor.org/packages/release/bioc/html/edgeR.html
RepeatMasker	Smit et al., 2015	http://www.repeatmasker.org/
Mouse Genomes Project	Nellåker et al., 2012 Keane et al., 2011 Yalcin et al., 2011	https://www.sanger.ac.uk/sanger/Mouse_SnpViewer/rel-1505
ENCODE	ENCODE Consortium	https://www.encodeproject.org/
Galaxy deepTools	Ramírez et al., 2014	https://usegalaxy.org
StringTie 1.3.3	Pertea et al., 2015	https://ccb.jhu.edu/software/stringtie/#pub
R	R Development Core Team, 2016	https://www.r-project.org/
Pfam	Finn et al., 2016	https://pfam.xfam.org/
Ensembl annotation	Zerbino et al., 2018	http://www.ensembl.org/index.html?redirect=no
Custom R code for data analysis (Github)	This paper	https://github.com/AFS-lab/Kazachenka-Bertozzi-et-al-2018

**Other**		

Streptavidin Sepharose High Performance	GE Healthcare	Cat#GE17-5113-01

### Contact for Reagent and Resource Sharing

Further information and requests for resources and reagents should be directed to and will be fulfilled by the Lead Contact, Anne C. Ferguson-Smith (afsmith@mole.bio.cam.ac.uk).

### Experimental Model and Subject Details

#### MICE

All mouse work was carried out in accordance with UK government Home Office licensing procedures (HO project license number: PC9886123). All experiments used C57BL/6J mice of both sexes. The methylation validation and expression experiments were performed on 8-10 week old mice. For the inheritance studies, mice were set up for breeding at 8 weeks of age and the F1 methylation level was assessed in ear notches from 10-12 day old pups. All mice were fed a standard chow diet *ad libitum* and housed in controlled temperature, humidity, and light-dark cycle (12h) conditions.

### Method Details

#### Tissue and B cell collection

Following dissection, somatic C57BL/6J tissues were snap frozen in liquid nitrogen and manually pulverized. B cells were isolated from fresh splenic tissues using the B Cell Isolation Kit (Miltenyi Biotec). Sperm collection and purification from cauda epididymis was done as described in Sharma et al. (2016).

#### DNA/RNA extraction and bisulfite conversion

30 ug of tissue (brain, kidney, liver, and spleen) was used for simultaneous purification of genomic DNA and total RNA using the AllPrep DNA/RNA Mini Kit (QIAGEN). During purification, RNA was treated with DNaseI using the RNase-Free DNase Set (QIAGEN). Ear notch DNA was purified using a standard phenol-chloroform extraction protocol. DNA was bisulfite treated using the two-step protocol of the Imprint DNA Modification Kit (Sigma).

#### DNA methylation analysis

Methylation quantification was carried out by pyrosequencing. Assays were designed using PyroMark Assay Design SW 2.0 (QIAGEN). Primers are provided in Table S4. Regions of interest were amplified from bisulfite converted DNA via PCR using biotinylated reverse primers and HotStarTaq DNA Polymerase (QIAGEN). The annealing temperature for PCR primers was optimized by gradient PCR. PCR conditions: 1) 95°C – 5 min; 2) 94°C – 30 s, optimized t°C – 30 s, 72°C – 55 s, 40 cycles; 3) 72°C – 5 min. PCR products were shaken at 1,400 rpm with Streptavidin Sepharose High Performance beads (GE healthcare) dissolved in binding buffer (10mM Tris-HCL pH7.6, 2M NaCl, 1mM EDTA, 0.1% Tween-20) for 20 min. The biotinylated strand was purified using the PyroMark Q96 Vacuum Workstation (QIAGEN). Sequencing primers were annealed to the template in annealing buffer (20mM Tris-acetate pH7.6, 2M magnesium acetate) at 85°C for 3 min. Sequencing was carried out on the PyroMark Q96 MD pyrosequencer (QIAGEN) using PyroMark Gold Q96 Reagents (QIAGEN).

#### Expression analysis

cDNA was synthesized using the RevertAid H Minus First Strand cDNA Synthesis Kit (Thermo Fisher Scientific). Q-PCR primers were designed using Primer3 software (Untergasser et al., 2012) and are listed in Table S4. cDNA was amplified using the LightCycler 480 SYBR Green I Master mix and LightCycler 480 Instrument (Roche). PCR conditions: 1) 95°C – 5 min; 2) 95°C – 10 s, 60°C – 10 s, 72°C – 10 s, 45 cycles; 3) 95°C - 5 s, 65°C - 1 min, 97°C - continuous ; 4) 40°C – 30 s. Relative cDNA abundance was calculated using the ΔCT method and normalized to housekeeping gene *β-actin*. The significance of correlations between expression and methylation levels was assessed by computing Pearson correlation coefficients followed by two-tailed p values in GraphPad Prism.

### Quantification and Statistical Analysis

#### Biased screen of polymorphic IAPs

Genomic coordinates of C57BL/6J-specific IAPs that are absent from the CAST/Eij genome were extracted from a published list of polymorphic ERVs (Nellåker et al., 2012). IAP coordinates were converted from the mm9 to the mm10 mouse genome assembly using liftOver (Hinrichs et al., 2006) and assigned to the nearest protein-coding gene from the Ensembl gene database (GRCm38) using Bedtools (Quinlan and Hall, 2010). DESeq2 and edgeR were used to identify differentially expressed (DE) genes for B and T cell samples. Due to the absence of strain-specific annotation of ncRNAs, they were removed from the analysis. Significant hits from both programs were used to compile the list of DE genes between the two strains. DE genes were overlapped with genes containing a polymorphic IAP insertion or having one nearby. The overlapping genes provided a list of candidate IAPs for visual assessment of methylation levels.

#### Genome-wide screen

The genome-wide screen for VM-IAPs used WGBS and WGoxBS datasets generated from B and T cells (16 datasets in total; accession number: GSE94676). WGBS and WGoxBS datasets were treated as biological replicates, as the WGoxBS protocol recognizes both methylated and hydroxymethylated DNA. The 5mC:5hmC ratio in the WGoxBS datasets used was 1:0.015. Each biological replicate consisted of pooled B or T cells for 4-5 individuals. Bedgraph files were used to extract methylation levels of IAP CpGs. Methylation of a CpG site is represented by two values, reflecting methylation levels of the sense and antisense strands. The average of 16 methylation values representing 8 distal CpGs from the 5′ or 3′ end of IAPs was calculated for each biological replicate to estimate a given IAP’s methylation level. To determine the magnitude of methylation variation at an IAP across the 16 biological replicates, the average methylation levels were sorted and the difference between the second highest and the second lowest values was used as a computational score for methylation variation (Figure S1). The analysis for 5′ and 3′ ends was done separately and subsequently overlapped. The threshold of variation used as a cut-off for the final list of VM-IAPs was determined by experimental assessment of the methylation variation of a subset of IAPs exhibiting a range of computational scores (Table S5). Differentially methylated regions between B and T cells were excluded from the final list of VM-IAPs since these constituted cell type-specific DMRs and hence did not fulfill the criteria of methylation consistency between tissues. An IAP was considered to be a DMR if its 8 highest and/or 8 lowest average methylation levels came from only one of the two cell types. The same process was carried out for sex-specific DMRs, but none were found.

#### Strain-specific polymorphism analysis

A catalog of structural variants across 18 inbred mouse strains generated for the Mouse Genomes Project (https://www.sanger.ac.uk/sanger/Mouse_SnpViewer/rel-1505) was used to quantify polymorphism of VM-IAP candidates (Keane et al., 2011, Yalcin et al., 2011).

#### Neighbor-joining tree analysis

The neighbor-joining tree of IAPs of the IAPLTR1_Mm subtype was built with Geneious 9.0.5 software using default parameters (Kearse et al., 2012, Faulk et al., 2013). IAP sequences were downloaded from the UCSC Table Browser and “+” strand sequences were used for antisense IAPs.

#### Co-variation analysis

Methylation levels at six VM-IAPs in 33 C57BL/6J mice were normalized to a given VM-IAP’s inter-individual methylation range. A normalized correlation matrix showing Pearson correlation coefficients as well as p values for the correlation of each VM-IAP pair was generated using GraphPad Prism. A Bonferroni-adjusted α value of 0.008 was used.

#### Generation of enrichment profiles

Histone modification and CTCF binding profiles were constructed using publicly available ENCODE datasets. Accession numbers for all ENCODE datasets used can be found in Table S6. Signal p value bigwig files were downloaded and analyzed using Galaxy deepTools (Ramírez et al., 2014).

#### Transcriptomic analysis

*De novo* transcriptomes were assembled using StringTie 1.3.3 software (Pertea et al., 2015). 12 RNA-seq datasets were used for *de novo* transcriptome assembly (three replicates of each cell type: female B cells, male B cells, female T cells and male T cells). Using Bedtools (Quinlan and Hall, 2010), the coordinates of the identified transcripts were overlapped with VM-IAP coordinates to identify transcripts initiated or terminated within VM-IAPs. Only VM-IAPs that overlapped transcripts in at least three biological replicates representing the same cell type and sex were further analyzed.

#### Inheritance analysis

The effect of parental methylation level on offspring methylation level was analyzed using REML-fitted linear mixed-effects models (LMMs) in R via the lmer() function in the lme4 package (Bates et al., 2015). The bisulfite pyrosequencing methylation levels of all individuals used for this analysis were averaged across the first four CpGs of the IAP LTR and run through a logit transformation before feeding into the model. The LMMs for each VM-IAP included maternal methylation level, paternal methylation level, and sex as fixed effects. Breeding pair as well as litter nested within breeding pair were treated as random intercept effects, accounting for the non-independence of siblings and littermates, respectively. Interaction between maternal and paternal methylation levels was originally assessed but no significant interaction was found. Parameter estimates, standard errors, and t values are reported in Figure S5B. By default, the reference intercept is selected alphabetically, in this case representing the estimate for female methylation level. To evaluate the significance of the fixed effects, p values were generated using the Satterthwaite approximation for degrees of freedom, applied by the lmerTest package in R (Kuznetsova et al., 2017). To account for multiple testing, q-values were generated using the Benjamini-Hochberg correction with a false discovery rate of 0.05 (Benjamini and Hochberg, 1995). To assess effect sizes, semi-partial R^2^ values for each fixed effect were calculated using the r2beta() function from the r2glmm package in R (Jaeger et al., 2017). For the repeated inheritance experiment on VM-IAP_Gm13849_, the mean methylation level of littermates was calculated for the first litter of five highly methylated and five lowly methylated mothers. Significance was assessed with a one-sided unpaired t test using GraphPad Prism.

##### Assembly of ERV coordinates

The RepeatMasker database was downloaded from the UCSC Table Browser to determine the genomic coordinates of ERV fragments (Karolchik et al., 2004). ERV fragments were separated into four groups according to RepeatMasker annotation (ERV1, ERVL, ERVK and IAPs) and assembled using Bedtools (Quinlan and Hall, 2010). The structure of ERV insertions was determined based on RepeatMasker insertion fragment annotation.

### Data and Software Availability

The accession number for the WGBS, WGoxBS, and RNA-seq datasets used in this study is GEO: GSE94676. Cell purification and DNA/RNA extraction protocols as well as data processing pipelines are available at the above accession site. The R code used for the algorithms and computation analyses in this study has been collated into the following Github file: https://github.com/AFS-lab/Kazachenka-Bertozzi-et-al-2018.

## Supplemental Information

Table S1. Validation of the Threshold for Genome-wide Identification of VM-IAPs, Related to Figure 1 and STAR Methods

Table S2. Additional VM-IAPs with Computational Variation Range ≥20.9375 (Computational Variation of IAP_Cdk5rap1) and ≤25, Related to STAR Methods

Document S1. Tables S3, S4, and S6

Table S5. Pyrosequencing Primers, Related to STAR Methods
