# Identification, Characterization, and Heritability of Murine Metastable Epialleles: Implications for Non-genetic Inheritance

**DOI:** 10.1016/j.cell.2018.11.017

**Published:** 2018-11-29

**Authors:** Anastasiya Kazachenka, Tessa M. Bertozzi, Marcela K. Sjoberg-Herrera, Nic Walker, Joseph Gardner, Richard Gunning, Elena Pahita, Sarah Adams, David Adams, Anne C. Ferguson-Smith

(Cell *175*, 1259–1271.e1–e13; November 15, 2018)

In the Introduction to this article, we inadvertently neglected to cite Faulk et al., 2013. This study carried out a phylogenetic analysis of the IAPLTR1_Mm subclass of IAP elements, which includes the *A^vy^* and *Cabp^IAP^* loci. The authors show that this family is divided into three clades and provide data suggesting that the younger clades may be enriched for inter-individual methylation variation. This was corrected online on November 7, 2018.
