# Supplementary material for: Identification, Characterization, and Heritability of Murine Metastable Epialleles: Implications for Non-genetic Inheritance
Source: Cell. 2018 Nov 15;175(5):1259–1271.e13. doi: 10.1016/j.cell.2018.09.043 (PMC6242299; doi:10.1016/j.cell.2018.09.043)
Supplement: Document S1. Tables S3, S4, and S6 [file mmc3.pdf]

Table S3. Validation of VM-ERV candidates – Related to STAR Methods

ERV coordinates	ERV class	Experimental variation
chr4:134159390-134164702	ERV1	24.00667
chr11:50445185-50448338	ERVK	19.77667
chr17:87825643-87826512	ERVK	16.93467
chr9:119768870-119769617	ERVL	15.22333
chr18:30266511-30272200	ERV1	14.86
chr5:89203776-89209330	ERVK	14.28778
chr8:14878801-14879350	ERVK	14.09889
chr17:80562592-80564295	ERVK	13.21778
chr12:73551335-73551655	ERVK	12.882
chr10:127890018-127890535	ERVK	11.88333
chr19:61199870-61200383	ERV1	11.79667
chr15:55871468-55877005	ERVK	11.08875
chr5:92357515-92358372	ERV1	11.01333
chr17:14094391-14099888	ERVK	9.892222
chr7:49752788-49752997	ERVL	9.565833
chr6:108708205-108708524	ERVK	8.795
chr1:167637978-167638332	ERVL	8.598889
chr1:134400075-134405591	ERVK	8.3605
chr8:4799593-4800785	ERV1	7.935
chr8:105499850-105500332	ERVL	7.896667
chr8:119889564-119897040	ERV1	7.4975
chr17:87877890-87878685	ERVL	7.489167
chr16:17554302-17555119	ERVK	6.620833
chr17:32369421-32376516	ERVK	6.611111
chr8:35109846-35110151	ERVL	6.454667
chr18:61215716-61216076	ERVL	6.312222
chr16:94066286-94066593	ERVL	6.2075
chr17:87877890-87878685	ERVL	5.36
chr8:114391107-114398258	ERVK	5.326667
chr1:89096764-89102314	ERVK	5.292
chr4:126625495-126626267	ERVK	5.098889
chr5:114879784-114882457	ERVK	5.072708
chr11:117174448-117174893	ERVK	4.914167
chr6:85965716-85966239	ERV1	4.45875
chr9:78521525-78522134	ERV1	4.078333
chr2:178604020-178604661	ERV1	3.736667
chr11:86811741-86816399	ERV1	3.661875
chr9:68801476-68801953	ERVK	3.283333
chr12:58306552-58307085	ERVK	3.133333
chr6:87119268-87119744	ERVK	2.969259
chr19:44254266-44254775	ERV1	2.813
chr16:87665081-87665615	ERV1	2.765333
chr18:49551002-49551504	ERVK	2.48
chr1:191896787-191897062	ERVL	1.941111

Table S4. Q-PCR primers – Related to STAR Methods

	**Forward Primer**	**Reverse primer**
Eps8l1_ex12	GAGTCCTCAGGCACCTCC	ACTTTGCACTTGGTTTGGGT
Eps8l1_ex45	TGTCAATCACCTGGTCACCT	TCAGTAGCATCTCCTGTGCC
Slc15a2_ex12	TGAGTCCAAGGAAACGCTCT	GAAGAGCTTCGGAGTTGACT
Slc15a2_ex57	TGAAGAGGAACATGCAGAGG	GCATAGCAGTCTTCGCCAAA
Slc15a2_ex910	GCTTCAGGAACCGTTCTGAG	CGTCCATAATGAGGTGCTTTGG
Slc15a2_ex1920	GCAGTGCATTGTGAAACGGA	CCCCGGTTGGTGATATTAGTG
2610035D17Rik	CCCAACAGTCACCCATCCAT	GTCTCCTCGGCCTTTCTCTT
Bmf	CCAGAGACTCTTTTACGGCAAC	TGTTGCGTATGAAGCCGATG
Bub1b	GTCTCTGGATCAAATTGGGACA	CCTTGCGTTCAATCCCTTCC

Table S6. ENCODE datasets – Related to STAR Methods

Experiment	BigWig file	Bed file	Target	Tissue
ENCSR000CBJ	ENCFF594EHQ	ENCFF258WBW	CTCF	kidney male adult (8 weeks)
ENCSR000CCB	ENCFF069PTO	ENCFF520LES	CTCF	ES-Bruce4
ENCSR000CBU	ENCFF691VDG	ENCFF371UBQ	CTCF	liver male adult (8 weeks)
ENCSR000CBV	ENCFF870YTE	ENCFF967TRS	CTCF	lung male adult (8 weeks)
ENCSR000CBI	ENCFF824BON	ENCFF447SUY	CTCF	heart male adult (8 weeks)
ENCSR677SIH	ENCFF571UHJ	ENCFF095TOF	CTCF	lung embryo (14.5 days)
ENCSR418SBY	ENCFF709NWF	ENCFF116ZIX	CTCF	lung postnatal (0 days)
ENCSR491NUM	ENCFF246CLY	ENCFF139WDG	CTCF	heart postnatal (0 days)
ENCSR143WOK	ENCFF349HWD	ENCFF694LLR	CTCF	kidney postnatal (0 days)
ENCSR985ZTV	ENCFF309UUX	ENCFF726SOK	CTCF	midbrain postnatal (0 days)
ENCSR150RGT	ENCFF665MXC	ENCFF636SDF	CTCF	hindbrain postnatal (0 days)
ENCSR041SMK	ENCFF112EXI	ENCFF310FWM	CTCF	liver postnatal (0 days)
ENCSR677HXC	ENCFF323HFE	ENCFF779QUY	CTCF	forebrain postnatal (0 days)
ENCSR397RHW	ENCFF900YUX	ENCFF449LBJ	CTCF	liver embryo (14.5 days)
ENCSR238ZCJ	ENCFF135WIS	-	H3K9me3	lung postnatal (0 days)
ENCSR341XCL	ENCFF098ALU	-	H3K9me3	liver postnatal (0 days)
ENCSR498EVD	ENCFF010BLJ	-	H3K9me3	kidney postnatal (0 days)
ENCSR000CFZ	ENCFF872YDS	-	H3K9me3	ES-Bruce4
ENCSR000CBF	ENCFF016YZA	-	H3K4me1	ES-Bruce4
ENCSR308GFM	ENCFF182IJL	-	H3K4me1	liver postnatal (0 days)
ENCSR674PZU	ENCFF233UNG	-	H3K4me1	kidney postnatal (0 days)
ENCSR000CFN	ENCFF587RBP	-	H3K27me3	ES-Bruce4
ENCSR973UGS	ENCFF930GQD	-	H3K27me3	liver postnatal (0 days)
ENCSR564JKR	ENCFF339BGN	-	H3K27me3	kidney postnatal (0 days)
ENCSR000CFO	ENCFF202RDN	-	H3K36me3	ES-Bruce4
ENCSR656AMS	ENCFF229JQH	-	H3K36me3	liver postnatal (0 days)
ENCSR362PBD	ENCFF147HVT	-	H3K36me3	kidney postnatal (0 days)
ENCSR000CGS	ENCFF342SOW	-	H3K9ac	ES-Bruce4
ENCSR966RAG	ENCFF139ZOZ	-	H3K9ac	liver postnatal (0 days)
ENCSR288OHO	ENCFF357TID	-	H3K9ac	kidney postnatal (0 days)
ENCSR000CDE	ENCFF646XOT	-	H3K27ac	ES-Bruce4
ENCSR616TJM	ENCFF734CDU	-	H3K27ac	liver postnatal (0 days)
ENCSR140YPL	ENCFF605ABI	-	H3K27ac	kidney postnatal (0 days)
ENCSR000CBG	ENCFF611GSQ	-	H3K4me3	ES-Bruce4
ENCSR653AVN	ENCFF587FZP	-	H3K4me3	liver postnatal (0 days)
ENCSR536ILV	ENCFF926CET	-	H3K4me3	kidney postnatal (0 days)
